# Transcriptomic signatures of classical monocytes reveal pro-inflammatory modules and heterogeneity in polyarticular juvenile idiopathic arthritis

**DOI:** 10.3389/fimmu.2024.1400036

**Published:** 2024-05-21

**Authors:** Bidossessi W. Hounkpe, Lucas P. Sales, Surian C. R. Ribeiro, Mariana O. Perez, Valéria F. Caparbo, Diogo Souza Domiciano, Camille P. Figueiredo, Rosa M. R. Pereira, Eduardo F. Borba

**Affiliations:** Hospital das Clínicas, Faculdade de Medicina, Universidade de São Paulo (HCFMUSP), Sao Paulo, Brazil

**Keywords:** polyarticular juvenile idiopathic arthritis, classical monocytes, transcriptomic, inflammation, autoimmunity

## Abstract

**Introduction:**

Polyarticular juvenile idiopathic arthritis (pJIA) is a childhood-onset autoimmune disease. Immune cells contribute to persistent inflammation observed in pJIA. Despite the crucial role of monocytes in arthritis, the precise involvement of classical monocytes in the pathogenesis of pJIA remains uncertain. Here, we aimed to uncover the transcriptomic patterns of classical monocytes in pJIA, focusing on their involvement in disease mechanism and heterogeneity.

**Methods:**

A total of 17 healthy subjects and 18 premenopausal women with pJIA according to ILAR criteria were included. Classical monocytes were isolated, and RNA sequencing was performed. Differential expression analysis was used to compare pJIA patients and healthy control group. Differentially expressed genes (DEGs) were identified, and gene set enrichment analysis (GSEA) was performed. Using unsupervised learning approach, patients were clustered in two groups based on their similarities at transcriptomic level. Subsequently, these clusters underwent a comparative analysis to reveal differences at the transcriptomic level.

**Results:**

We identified 440 DEGs in pJIA patients of which 360 were upregulated and 80 downregulated. GSEA highlighted TNF-α and IFN-γ response. Importantly, this analysis not only detected genes targeted by pJIA therapy but also identified new modulators of immuno-inflammation. *PLAUR*, *IL1B*, *IL6*, *CDKN1A*, *PIM1*, and *ICAM1* were pointed as drivers of chronic hyperinflammation. Unsupervised learning approach revealed two clusters within pJIA, each exhibiting varying inflammation levels.

**Conclusion:**

These findings indicate the pivotal role of immuno-inflammation driven by classical monocytes in pJIA and reveals the existence of two subclusters within pJIA, regardless the positivity of rheumatoid factor and anti-CCP, paving the way to precision medicine.

## 1 Introduction

Juvenile idiopathic arthritis (JIA) is a heterogeneous group of chronic systemic inflammatory disease that affects children and adolescents under 16 years (1). According to the International League of Associations for Rheumatology (ILAR), JIA is categorized into six subtypes based on the number of affected joints in the first 6 months of disease and the presence of extra-articular involvement (2). Among the subtypes of JIA, polyarticular JIA (pJIA) is characterized by inflammatory arthritis affecting five or more joints (3). Despite its unknown etiology, pJIA is an autoimmune disease mediated by lymphocytes arising from the dysregulation of the adaptive immune system. Accordingly, autoantigens derived from cartilage activate self-reactive T cells, including Th1 and Th17 cells, leading to the production of pro-inflammatory cytokines such as IFN-γ and IL-17 (4). Conversely, the inhibition of regulatory T cells (Treg) leads to a decrease in anti-inflammatory cytokine IL-10 production, resulting in loss of immune tolerance. The imbalance between self-reactive Th1/Th17 cells and Treg cells causes the breakdown of T-cell tolerance to autoantigens, contributing to synovial inflammation (4). In addition to the involvement of adaptive immune cells, myeloid cells, particularly monocytes and dendritic cells, also play a crucial role in the regulation of immuno-inflammation in pJIA.

Human monocytes can be categorized into three distinct subtypes: classical (CD14++CD16−), intermediate (CD14++CD16+), and non-classical (CD14+CD16++). Monocytes can exhibit a rapid innate effector function, demonstrating the capability to initiate and drive inflammation (5). Upon activation, classical monocytes secrete elevated levels of pro-inflammatory cytokines including TNF-α, IL-1β, and IL-6 (5). Previous studies have demonstrated that the composition of blood classical monocytes is elevated in adult-onset arthritis, such as rheumatoid arthritis, compared to healthy individuals (6). Recently, it has been shown that synovial monocytes and macrophages from childhood-onset arthritis patients are polarized and contribute to chronic inflammation via the IL-6/JAK/STAT signaling pathway (7). Upon stimulation of peripheral blood mononuclear cells (PBMCs) by IL-6 and IFN-γ, classical monocytes isolated from treatment-naive pJIA exhibit an increased IFN-γ signaling compared to healthy samples (8). This IFN-γ response seems to be heterogeneous between patients, suggesting the contribution of classical monocytes to the heterogeneity observed within JIA subtypes.

Gene expression signatures from PBMC isolated from children with recent-onset pJIA has been utilized to stratify these patients in cluster with distinct transcriptomic profile, revealing the heterogeneity between pJIA (9). However, the contribution of specific blood cell types to this heterogeneity remains unclear. Recently, using RNA sequencing, we demonstrated that classical monocytes isolated from rheumatoid arthritis (RA) patients are involved with the excessive activation of immuno-inflammatory pathways and bone erosion observed in this disease (10). Nevertheless, the role of the subtype of classical monocyte in pJIA is still uncertain. Therefore, the aim of the present study was to assess the transcriptomic profile of classical monocyte in adult pJIA patients compared to health subjects and to explore their potential contribution to the heterogeneity of pJIA.

## 2 Materials and methods

### 2.1 Study population

A total of 18 premenopausal pJIA women regularly followed in the Rheumatology Outpatient Clinic at the Hospital das Clinicas da Universidade de São Paulo were recruited for this study. All patients fulfilled the ILAR 2001 classification criteria for pJIA (2). Patients were excluded if they had metabolic bone disease (e.g., rickets, primary hypoparathyroidism, osteomalacia, and Paget’s disease), ii) use of any medication interfering with bone metabolism (e.g., prednisone doses higher than 7.5 mg/day, bisphosphonates, and bone-targeted monoclonal antibodies), iii) other autoimmune disease, or iv) pregnancy or lactation. Premenopausal status was assessed through a self-reported information as previously described (10). In addition, clinical and transcriptomics data of 17 age- and body mass index-matched healthy subjects were extracted from our previously published database (10). The inclusion and exclusion criteria were carefully delineated for the healthy controls group. Only female participants without osteometabolic diseases were eligible for inclusion in the study. Additionally, individuals with any autoimmune and/or non-communicable chronic diseases, those who were pregnant, had bone metabolism disorders (like hyperparathyroidism, Paget’s disease, and bone dysplasia), had neoplasms, or were using medications that could disrupt bone metabolism were excluded (10). The study was approved by the local Ethics Committee from Sao Paulo University-CAPPesq (#51178115.1.0000.0068). All participants gave written informed consent, in accordance with the principles of the Declaration of Helsinki.

### 2.2 Clinical and laboratory assessments

Demographic and clinical data were obtained through interviews and electronic medical records, including age, weight, height, race, coexisting chronic diseases, time from pJIA onset, and treatment data (1, 10). Erythrocyte sedimentation rate (ESR), C-reactive protein (CRP), rheumatoid factor (RF), and anti-cyclic citrullinated peptide (anti-CCP) antibodies were measured using standard automated methods. Disease activity was assessed in a standard manner using the Simplified Disease Activity Index (SDAI): >3.3 and ≤11 (low disease activity), >11 and ≤26 (moderate disease activity), and >26 (high disease activity) (1, 10).

### 2.3 Classical monocytes isolation from human peripheral blood

Peripheral blood samples were obtained from pJIA patients through venous puncture into BD Vacutainer System^®^ vacuum tubes containing K3EDTA anticoagulant (0.15 mg/mL, Becton Dickinson, USA). Subsequently, PBMCs were isolated using Ficoll-Hypaque density gradient centrifugation and dextran sedimentation. The PBMCs were then labeled with biotin-conjugated monoclonal antibodies cocktail targeting antigens not expressed in human monocytes, utilizing the commercial Pan Monocyte Isolation kit (Myltenyi^®^, Germany). Following this step, microbeads conjugated to anti-biotin monoclonal antibodies were introduced, enabling magnetic separation and isolation of a pure monocyte population in the filtrate. Monocytes were further distinguished by labeling with CD16+ antibodies, facilitating the separation of subpopulations. The CD14++CD16− monocytes were isolated via negative selection.

### 2.4 RNA-sequencing

The RNA extraction process for classical monocytes was performed using the RNeasy Plus Mini kit (Qiagen^®^). Subsequently, the quality of the extracted RNA was assessed, ensuring RNA integrity number (RIN) of at least 7, evaluated via the ScreenTape method using Bioanalyzer 2100 (Agilent^®^). To construct the RNA library, the Quant-seq^®^ 30 mRNA-Seq Library Prep Kit (Lexogen^®^) was employed, followed by sequencing on an Illumina HiSeq 2500 platform (Illumina^®^).

### 2.5 RNA-sequencing data processing

The initial processing of RNA-sequencing data involved stringent quality control measures using FastQC tool and comparing the fraction of housekeeping genes detected across samples (11). Only high-quality raw sequencing data were included for subsequent analysis after undergoing trimming to eliminate sequences of low quality and poly-A sequences. This trimming process was executed using the BBDuk tool. Subsequently, the resulting reads were aligned to the human reference genome (GRCh38/Ensembl) using the STAR aligner (12). The generated read count matrices were then imported into R version 4.1 for downstream analyses.

### 2.6 Differential expression analysis

Differential expression analysis was performed by comparing pJIA patients with healthy controls utilizing DESeq2 and leveraging the negative binomial distribution (13). Differentially expressed genes (DEGs) were identified based on a fold change threshold of 2 and controlling the false discovery rate (FDR) using the Benjamini–Hochberg method with a cutoff set at <0.05. A list of genes associated with pJIA was extracted from the OpenTarget platform, which integrates public data relevant to the association between targets and diseases, including data from genetics, expression analysis, drugs, and animal models. The log_2_ fold changes and standard errors of these genes were plotted. Volcano plots and heatmaps were designed using ggplot2 package and the ComplexHeatmap package (14). Furthermore, we performed a Pearson’s correlation analysis to examine the relationship between the fold changes derived from comparing pJIA vs. control groups and the fold changes observed in rheumatoid arthritis (RA) patients vs. control data. The fold changes of RA vs. control were retrieved from our previous study deposited on ArrayExpress (E-MTAB-13361) (10). These RA patients fulfilled the classification criteria for RA defined by the American College of Rheumatology/European Alliance of Associations for Rheumatology in 2010. The criteria for inclusion and exclusion have been described in our previous study (10).

### 2.7 Silhouette analysis and K-means clustering

Normalized counts derived from patients’ samples were used to create a subset dataset comprising 1,000 highly variable genes. Subsequently, samples underwent clustering employing the k-means clustering algorithm. Silhouette analysis was conducted to determine the optimal number of clusters. pJIA patients were categorized into two subgroups based on their assigned clusters. The comparison between these clusters and the control group was performed through a likelihood ratio test (LRT) to discern differences. Pairwise comparison was specifically performed within the subset of pJIA patients clusters, to identify DEGs using the Wald test, considering a fold change threshold of 2 and a FDR controlled using the Benjamini–Hochberg method, with a cutoff set at <0.05.

### 2.8 Features selection

Recursive feature elimination (RFE) was performed using support vector machine (SVM) model with linear kernel and 5-thold cross-validation as implemented in scikit-learn. Features (genes) were eliminated recursively, and the optimal number of features was based on min accuracy metric. The most informative features were then selected based on their weight.

### 2.9 Gene sets enrichment analysis and network reconstruction

Robust gene set enrichment analyses were conducted using GSEA and single-sample GSEA (ssGSEA) tools (15, 16). A significance threshold of FDR < 0.1 was applied. The network, formed by the significant GSEA pathways and their interconnected shared genes, was reconstructed in Cytoscape (17). A network connectivity analysis was conducted to identify hub genes based on their centrality (degree centrality).

### 2.10 Correlation analysis

Pearson correlation analysis was performed to decipher the relationship between clinical and laboratorial characteristics, and the normalized counts of genes associated with activated pathways. p-values < 0.05 were considered significant.

### 2.11 Statistical analysis

Parametric assumptions were assessed, and quantitative variables were compared using independent t-test or Mann–Whitney test, as appropriate. Percentages were analyzed by chi-square (χ^2^) or Fisher’s exact test. Data were presented as mean ± standard deviation (SD) or median (first and the third quartiles) for continuous variables and number (percentage) for categorical variables. Statistical analyses were performed with SPSS software (version 20) and R (version 4.1). The significance level was set at two-sided p-value < 0.05.

## 3 Results

### 3.1 Clinical and demographic features

The demographic and clinical characteristics of pJIA and healthy groups are demonstrated in **Table 1**. The median age at diagnostic of patients was 9.5 years, and the median disease duration was 25 years. C-reactive protein (CRP) showed no statistically significant difference between pJIA and healthy, although pJIA patients exhibited wider first and third quartiles intervals (0.9–6.8 mg/L and 0.8–2.8 mg/L for pJIA and healthy controls, respectively), indicating an heterogeneity in the pJIA group. The ESR was significantly higher in pJIA patients compared to healthy controls. RF and anti-CCP were positive in 28% and 50% of pJIA patients, respectively. pJIA patients present low disease activity based on CDAI (9.6 ± 7.4) and SDAI (10.0 ± 7.3) score. Three patients received glucocorticoid, one of whom also used bDMARD. Upon comparison with all other patients, no statistically significant difference in disease activity was observed (SDAI 11 ± 7.1 for patients using glucocorticoid and/or bDMARD, compared to 9.7 ± 7.5 for all other patients; p-value = 0.7).

**Table 1:** Demographic and clinical characteristics.

Variables	pJIA (n=18)	Healthy controls (n=20)	*p*
Age, years	32.7 ± 7.3	37.1 ± 6.2	0.068
BMI, kg/m^2^	24.1 ± 4.1	25.1 ± 5.1	0.487
Race, no. (%)			
Caucasian	9 (50)	4 (24)	0.105
Non-Caucasian	9 (50)	13 (76)	
Disease duration, years (Q1–Q3)	25.0 (15.2–31.7)	–	–
Age at diagnosis, years (Q1–Q3)	9.5 (3.0–14.0)	–	–
ESR, mm (Q1–Q3)	14.5 (9.75–31)	7 (5–13)	**0.016**
CRP, mg/L (Q1–Q3)	3.3 (0.9–6.8)	1.0 (0.8–2.8)	0.162
Glucocorticoid, n (%)	3 (17)	–	–
Glucocorticoid, mg/day	5 ± 0	–	–
csDMARD, n (%)	14 (78)	–	–
bDMARD, n (%)	1 (6)	–	–
Comorbidities			
Hypertension, n (%)	1 (6)	–	–
Diabetes mellitus, n (%)	0 (0)	–	–

Results are expressed as median (first and the third quartiles), or n (%). BMI, body mass index; CRP, C-reactive protein; ESR, erythrocyte sedimentation rate; csDMARD, conventional synthetic disease-modifying antirheumatic drugs; bDMARD, biologic disease-modifying antirheumatic drugs. Q1, first quartile; Q3, third quartile.

Statistically significant p-values are highlighted in bold.

### 3.2 Classical monocytes exhibit an inflammatory phenotype in pJIA patients

**Figure 1:**
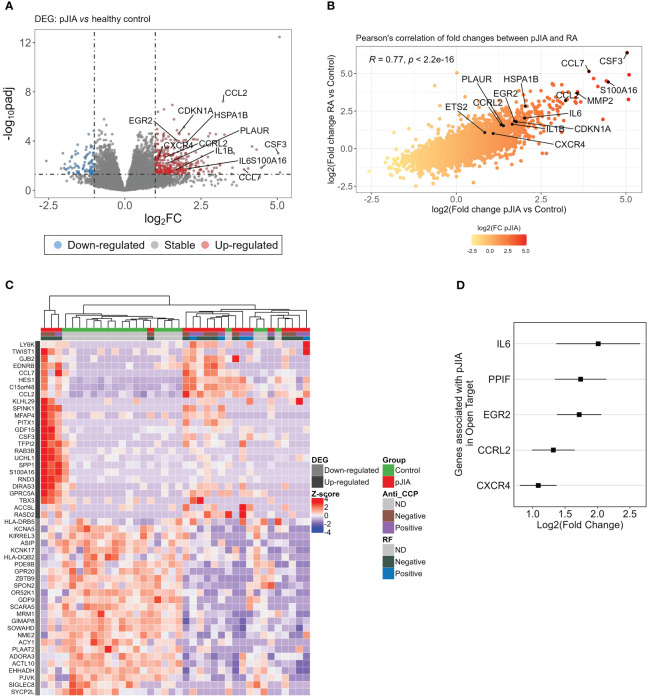
Differential expression analysis of classical monocytes’ genes in pJIA reveals similarities with RA. **(A)** Volcano plot showing DEGs identified from the comparison of pJIA vs. control group. A total of 440 DEGs were highlighted, of which 360 were upregulated (red dots) and 80 downregulated (blue dots). DEGs were identified based on a fold change of 2 and a Benjamini–Hochberg false discovery rate (FDR) using a cutoff set at <0.05. **(B)** Pearson’s correlation between the fold changes of pJIA and RA, both compared with control group, revealed high positive correlation. Genes involved in inflammatory mechanisms are highlighted in the plot. **(C)** Heatmap showing the expression profiles of the top 50 DEGs and unsupervised clustering of sample. This reveals a clear stratification of pJIA and controls in different clusters and a heterogeneity within the pJIA group. Hierarchical clustering of samples was performed based on the Euclidean distance calculated from the normalized and scaled expression. **(D)** Forest plot showing genes previously associated with pJIA in Open Target database. Log2 of fold change and standard errors are plotted. DEGs, differentially expressed genes; pJIA, polyarticular juvenile idiopathic arthritis; padj, adjusted p-value; RA, rheumatoid arthritis.

To identify the expression profile exhibited by classical monocytes isolated from pJIA patients, gene expression levels of these patients were compared with a healthy control group. We identified 440 DEGs in pJIA patients of which 360 were upregulated and 80 downregulated (**Figure 1A**, **Supplementary Table S1**). From the list of DEGs, we observed certain similarities between patients with pJIA and RA (10), particularly the upregulation of inflammatory mediators highlighted in **Figures 1A, B**. We performed a Pearson’s correlation analysis, revealing a strong positive correlation (**Figure 1B**) between the fold changes observed in pJIA vs. control and RA vs. control previously. The fold changes of RA data were extracted from our previous study deposited on ArrayExpress (E-MTAB-13361) (10) and reused in the present study. Several pro-inflammatory mediators such as *IL1B*, *IL6*, *CCL2*, *CCL7*, and *PLAUR* were upregulated in pJIA and are relevant in the pathogenesis of pJIA.

Heatmap and hierarchical clustering using the top 50 ranked DEGs shows the pattern of expression and a clear stratification of pJIA and controls samples in distinct clusters (**Figure 1C**). Consistent with the role of inflammation in the pathogenesis of pJIA, we observed an enrichment of genes associated with inflammation (*CCL7*, *CCL2*, *CSF3*, and *SPP1*) among the top upregulated DEGs. We further investigated whether any of the identified DEGs had been previously associated with pJIA by integrating our list with gene associated with pJIA in Open Target database (18). Interestingly, several upregulated genes (*IL6*, *PPIF*, *EGR2*, *CCRL2*, and *CXCR4*), but not downregulated genes, have been previously associated with pJIA (**Figure 1D**).

### 3.3 Classical monocytes exhibit TNF-α and IFN-γ responses in pJIA

**Figure 2:**
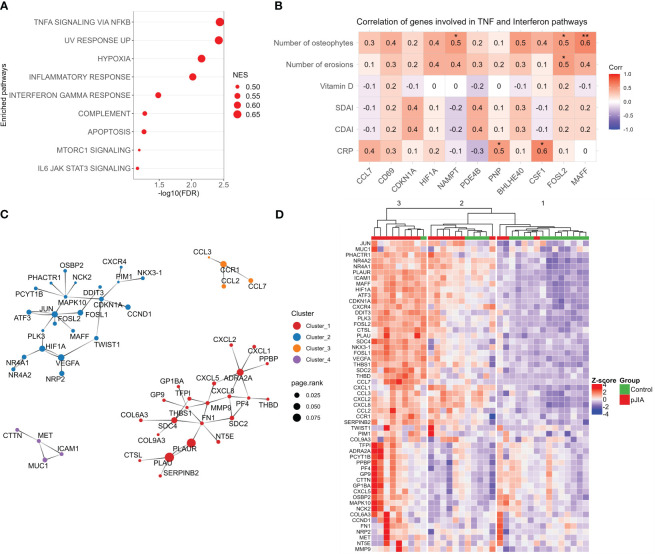
Functional gene set enrichment analysis and pJIA-associated gene modules. **(A)** Dot plot of functional analysis performed using the hallmark gene sets (GSEA) shows the enrichment of pathways associated with the activation of immuno-inflammation. Dot size represents the normalized GSEA enrichment score (NES), and the X-axis indicates the FDR. Pathways with FDR of <0.1 was considered significant. **(B)** Correlation analysis between clinical and laboratorial characteristics with normalized gene expression. Only genes associated with TNF-α and IFN-γ were included. Red color indicates positive correlation and blue, negative correlation. *p-value. **(C)** Protein–protein interactions network reconstructed with upregulated genes. Four highly connected modules are represented. Colors indicate each module (cluster), and the dot size indicates the page rank score of genes belonging to these modules. **(D)** Heatmap showing the expression profiles of genes belonging to modules identified in panel **(C)**. Unsupervised clustering of sample shows a stratification of pJIA and controls in three different clusters. Hierarchical clustering of samples was performed based on the Euclidean distance calculated from the normalized and scaled expression. Numbers (1, 2, and 3) on top of the dendrogram indicate the clusters. GSEA, gene set enrichment analysis; FDR, false discovery rate; NES, normalized enrichment score.

To gain more insights into the activated processes in classical monocytes of pJIA, a functional gene set enrichment analysis (GSEA) was conducted using all the DEGs identified in pJIA. This analysis confirmed an activation of biological processes associated with inflammation in pJIA. Particularly, GSEA highlighted an overrepresentation of TNF-α, interferon gamma, and IL6/JAK/STAT3 signaling in pJIA (**Figure 2A**). Notably, this analysis not only detected genes and pathways already targeted by pJIA therapy but also identified new modulators of immuno-inflammation in these patients (**Supplementary Figure S1**). Network analysis of the activated GSEA pinpointed *PLAUR*, *IL1B*, *IL6*, *CDKN1A*, *PIM1*, and *ICAM1* as relevant hub genes and drivers of chronic hyperinflammation (**Supplementary Figure S1**). Furthermore, TNF alpha signaling via NFKB was identified as the most interconnected pathway (**Supplementary Figure S1**). Notably, positive correlations were obtained between pJIA clinical and laboratory characteristics and gene expression levels of several TNF-α and IFN-γ response genes: the number of osteophytes with expression of *MAFF*, *FOSL2*, and *NAMPT*; the number of bone erosions with *FOSL2*; and C-reactive protein levels with PNP and CSF1 (**Figure 2B**).

### 3.4 Protein–protein interactions network reconstruction of upregulated genes revealed relevant pro-inflammatory modules

Protein–protein interactions (PPI) are fundamental for homeostasis, and their dysfunction can be associated with disease states (19). To identify PPI modules that emerge from upregulated DEGs, we reconstructed a PPI network using a network expanding approach. After extracting the high connected networks, we identified four interaction modules (**Figure 2C**). Modules 1–3 are enriched in cytokines/cytokines receptors and others inflammatory mediators, while module 4 includes the adhesion protein *ICAM1* and the tyrosine-protein kinase *MET*.

After identifying the connected modules associated with pJIA, we investigated the expression patterns of associated genes in patients and healthy control groups. The heatmap in **Figure 2D** shows the expression profile and unsupervised clustering of pJIA and control samples in three distinct clusters (**Figure 2D**). Detailed analysis of theses clusters reveals the predominant stratification of pJIA patients in clusters 2 and 3, and healthy controls in cluster 1. Notably, cluster 3 is predominantly composed of pJIA (eight out of nine samples), while clusters are composed of a mixture of pJIA and control samples. Dendrogram analysis indicates that cluster 2 is closer to cluster 1 (healthy controls) than cluster 3. These findings suggest that the pJIA patient group is a heterogeneous population with varying levels of inflammation.

### 3.5 Unsupervised clustering of pJIA reveals heterogeneity in inflammatory response

**Figure 3:**
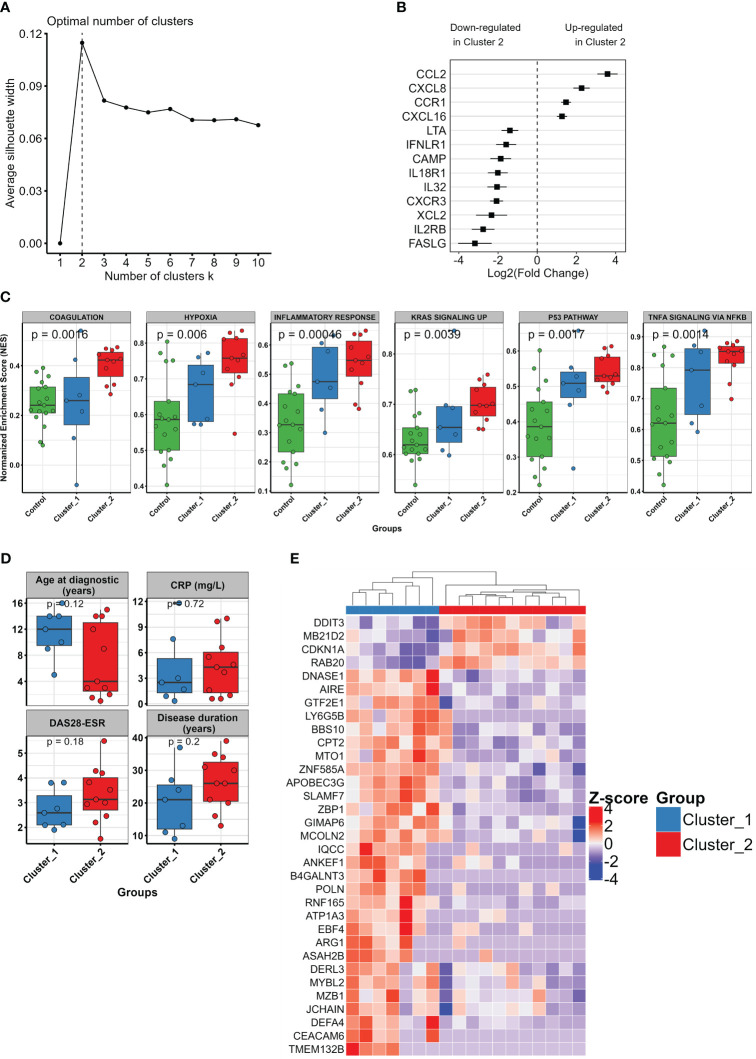
Unsupervised learning and clustering unveil the heterogeneity in pJIA. **(A)** Optimal number of clusters indicated by silhouette analysis. Based on average silhouette score, the vertical dashed line shows two clusters as the optimal cluster number for pJIA accurate stratification. **(B)** Forest plot showing the differential pattern of cytokines and their receptors in the identified pJIA clusters. Log2 fold change of cluster 2 vs. cluster 1 and standard errors are plotted. **(C)** Boxplot showing varying activation levels of inflammatory pathways between control group (green), cluster 1 (blue), and cluster 2 (red). Points indicate the single sample GSEA score of each sample. p-values from Kruskal–Wallis test are shown. **(D)** Boxplot showing the comparison of clinical characteristics between cluster 1 and cluster 2. Mann–Whitney test was performed. **(E)** Heatmap revealing the pattern of expression of informative features selected with recursive feature elimination approach using SVM with a linear kernel. A total of 33 genes were identified, of which four were upregulated and 29 downregulated in cluster 2. GSEA, gene set enrichment analysis; SVM, support vector machine.

In order to unveil the heterogeneity among pJIA patients, we use an unsupervised learning approach to stratify these patients in clusters based on their similarities at transcriptomics levels. Silhouette score analysis indicated that two clusters represent the optimal number of clusters for stratifying our pJIA samples (**Figure 3A**). Then, we used k-means to stratify pJIA patients in two different groups (clusters 1 and 2), each characterized by unique transcriptomic profile. DEGs analysis between cluster 2 and cluster 1 identified 432 DEGs, of which 129 were upregulated and 303 downregulated (**Supplementary Table S2**). Unsupervised clustering of pJIA patients based on the top 50 ranked DEGs revealed a stratification of pJIA in two homogenous clusters (**Supplementary Figure S2**). Cytokine and their receptors analysis shows different patterns between clusters (**Figure 3B**). While *CCL2*, *CXCL8*, *CCR1*, and *CXCL16* were upregulated in cluster 2, *LTA*, *INFLR1*, *CAMP*, *IL18R1*, *IL32*, *CXCR3*, *XCL2*, *IL2RB*, and *FASLG* were highly expressed in cluster 1. These findings confirm the presence of two population pJIA with varying inflammatory pathways. We then performed a GSEA analysis, which indicated that cluster 2 contains the most inflamed pJIA patients, exhibiting an enrichment of pro-inflammatory pathways (**Supplementary Figure S3**). A comparison of single sample GSEA score (ssGSEA) between pJIA clusters and control group revealed a gradual increase in the activity of inflammatory pathways from the control group to cluster 1, and then cluster 2 (**Figure 3C**). This result supports that patients within cluster 1 exhibit an intermediate level of inflammation. To determine whether pJIA clusters also exhibit distinct clinical characteristics, we compare clinical and laboratorial parameters between cluster 2 and cluster 1. We did not identify any statistically significant difference between clusters (**Figure 3D**). Interestingly the median age at diagnostic is higher in cluster 1 than in cluster 2, while CRP levels, DAS28-ESR, and disease duration show an increasing trend although not significant.

Finally, to select a set of informative genes that are capable of stratifying accurately pJIA patients in two different clusters, we perform a recursive feature elimination using SVM with a linear kernel. This approach identified 33 genes, of which four were upregulated and 29 downregulated in cluster 2. Using unsupervised clustering of pJIA based on the expression pattern of these genes, we observed a clear difference of gene expression pattern between cluster 1 and cluster 2, leading to a subsequent stratification in two distinct groups (**Figure 3E**).

## 4 Discussion

The present study demonstrated the activation of immuno-inflammatory pathways and associated genes in adult pJIA patients. Our data revealed that classical monocytes are activated by TNF-α and IFN-γ in pJIA. Although patients exhibited a low disease activity, our approach uncovered a heterogeneity in pJIA and identified two distinct patients’ groups. This suggests that transcriptomic data from classical monocytes can inform us about the heterogeneity observed between pJIA patients. Using unsupervised learning and feature selection approaches, we identified genes that are capable of stratifying patients in groups that exhibited unique transcriptomic patterns.

Activated classical monocytes have been associated with RA pathogenesis and bone erosion mechanisms (10, 20). However, their involvement to pJIA pathophysiology is not clear. Here, we analyzed a group of adult patients with a long disease duration (median of 25 years). Adult pJIA patients are known to share several clinical presentations with RA patients, such as symmetrical erosive disease of hands and wrists joints (1, 21) and an increase in pro-osteoclastogenic mediators (RANK, RANKL, TNF-α, and IL-6) (10, 22). Our results support these observed similarities and reveal, at transcriptomic level, a high correlation between the magnitude of the fold changes in pJIA and RA patients, compared with healthy controls. Furthermore, our findings highlight the importance of pro-inflammatory mediators such as *IL1B*, *IL6*, *CCL2*, *CCL7*, and *PLAUR* in the pathogenesis of both diseases. In pJIA patients group, classical monocytes exhibit an activation of pro-inflammatory genes induced by TNF-α and IFN-γ (*CCL7*, *CCL2*, *CSF3*, *SPP1*, *IL1B*, *IL6*, *MAFF*, *FOSL2*, *FOSB*, *NAMPT*, *CDKN1A*, *PIM1*, *ICAM1*, and *PLAUR*), two well-known mediators of immuno-inflammation in autoimmune and chronical inflammatory diseases (23, 24) and targeted by several anti-inflammatory drugs.

Several of these pro-inflammatory genes are associated with critical mechanisms involved in pJIA disease progression. Osteopontin, encoded by SPP1, is a multifunctional protein associated with various chronic inflammatory diseases, including RA (25, 26), systemic sclerosis (27), and inflammatory bowel disease (28), and have been correlated with disease severity (29). Osteopontin is an adhesion molecule involved in osteoclasts attachment to mineralized bone matrix and the upregulation of IFN-γ, following a positive feedback loop after its induction by IFN-γ signaling (30). In pJIA patients, the upregulation of SPP1 suggests the involvement of classical monocytes in mechanisms of bone resorption. Accordingly, we previously demonstrated that activated classical monocytes exhibit increased activation of pathways associated with bone erosion and a downregulation of pathways related to bone formation impairment (10).

Among the list of genes regulated by TNF-α, we also identified the transcription factors, *MAFF*, *FOSB*, and *FOSL2*. MAFF is a member of the MAF family of bZIP transcription factors and its expression is induced by pro-inflammatory cytokine (31). MAFF is shown to be a direct regulator of the chemokine CXCL1 and the cytokine CSF3 (31) that are upregulated in pJIA’s classical monocytes. This study observed a positive correlation between the expression levels of MAFF, FOSL2, and the number of osteophytes identified in pJIA patients, and between FOSL2 and the number of bone erosions identified in pJIA. Furthermore, FOSL2 and MAFF belongs to the same PPI module of pJIA (**Figure 2C**) and appear in a direct interaction, indicating that MAFF and FOSL2 contribute together to the regulators of inflammation in classical monocytes derived from pJIA. Indeed, FOSL2 is a member of AP1 heterodimers transcription complex formed by proteins of the JUN, FOS, ATF, and MAF family (32). Interestingly, JUN and ATF3 are also upregulated in pJIA compared with healthy group (**Supplementary Table S1**). In a Fosl2 overexpressing mice model, AP1 has been shown to promote systemic autoimmunity and multiple organ inflammation by repressing regulatory T cells development (32). Several immuno-inflammatory genes upregulated in pJIA such as IL6, known be involved in osteoclast formation, have been associated with bone erosion associated in classical monocytes from RA (10). In RA, an imbalance between RANKL and osteoprotegerin leads to increased osteoclast formation and bone resorption. AP1 is one of the key transcription factors activated by RANKL/TRAF signaling, and his crucial role has been pinpointed in osteoclast development. Consequently, the inactivation of Fos causes severe osteopetrosis due to the absence of osteoclasts (33, 34). The pro-inflammatory profile described in our results indicates that upon egress to inflamed synovial, classical monocytes derived from pJIA are capable to contribute to the mechanisms of imbalanced bone resorption. Consistently, these classical monocytes exhibit high expression of two adhesion molecules, SPP1 and ICAM1, necessary to their adhesion to blood vessels and to mineralized bone matrix (35).

When analyzing the topology of the network of activated pathways in pJIA, we identified *PLAUR*, *IL1B*, *IL6*, *CDKN1A*, *PIM1*, and *ICAM1* as the most relevant hub genes. IL1B, IL6, TNF, and IFN are critical for sustaining inflammation in pJIA. These cytokines are responsible for activating the expression of pro-inflammatory mediators during chronic inflammation. It has been demonstrated that IL1B induces the expression of PLAUR, gene encoding the urokinase plasminogen activator receptor (uPAR), and PLAU (urokinase plasminogen activator; uPA) (36, 37). Both PLAUR and PLAU are upregulated in pJIA. In a collagen-induced arthritis mice model, uPAR-mediated proteolysis of pro-uPA into uPA promotes the inflammation of joint that cumulates to arthritis progression (38). In addition, the increased uPA catalytic activity promotes synovial tissue destruction in RA (39), and soluble uPAR levels have been associated with disease activity in early untreated RA and reflects joint damage at later stages (40). Following simulation by IFN-γ, classical monocytes and CD4 T cells respond more strongly in treatment-naive pJIA patients than in the control group (8). High heterogeneity of expression surface markers across these patients have been observed upon the stimulation (8), supporting the role of IFN-γ response in the heterogeneity observed within pJIA. In this study, we highlighted the contribution of cytokines and their receptors, associated with TNF and IFN response, to the variability depicted in heatmap and unsupervised clustering.

Current JIA classification system lacks precision and fails to stratify patients in distinct homogeneous categories that reflect their clinical and biologic characteristics, treatment responses, disease courses, and outcomes (41). Consistent with these observations, our results spot a heterogeneity, driven by immuno-inflammation activation, within pJIA subtypes at transcriptomic levels. Through unsupervised clustering analysis, we demonstrated that the gene expression signature of classical monocytes can be utilized not only to assess inflammatory levels in pJIA but also to classify these patients into homogeneous biological subsets. Previous studies have indicated that biomarker profiles in JIA do not consistently align with patient categories (9, 42, 43), highlighting the necessity to integrate panel of clinical and biomarker features to refine classification systems, define therapies, and predict and enhance outcomes (41). Our feature selection approach identified a panel of 30 genes capable of stratifying pJIA patients in two distinct groups. Although we did not observe any significant differences in clinical characteristics, CRP levels, DAS28-ESR, and disease duration showed an increasing trend, which align with the increased inflammation levels observed in cluster 2 compared to cluster 1 and the control group (**Figure 3C**).

Our study has limitations that need to be acknowledged. We analyzed a relatively small sample of pJIA group. pJIA belongs to rare disease category, limiting the recruitment of large population of adult patients that fulfilled our inclusion and exclusion criteria. Despite the limited samples size, we did identify relevant features that clearly stratified pJIA patients and healthy controls in distinct clusters, validating our approach. Furthermore, pJIA patients included in this study exhibited a low disease activity. Thus, caution is warranted when extrapolating the results to patients with high disease activity. Our findings consistently identified relevant compartments of chronic inflammation in pJIA, strongly supporting the role of classical monocytes in pJIA independent of the varying inflammatory profile that can be observed within JIA subtype. Finally, we did not validate our findings at protein. Giving the potential disparity between gene expression and protein levels within the same tissue, it is essential to interpret our findings with this caveat in mind.

In conclusion, we identified crucial modulators of immuno-inflammation in pJIA, pointing classical monocyte as relevant cell type associated with pJIA disease mechanisms. We also demonstrated the pivotal role of TNF-α and IFN-γ signature in inflammation driving by these monocytes in pJIA. Our unsupervised learning approach revealed the existence of two subclusters within pJIA, regardless of their rheumatoid factor and anti-CCP positivity. These findings can lay the groundwork for precision medicine in the tailored management of these patients. Our list of identified genes also revealed modulators, previously unassociated with pJIA, that could serve as potential classifier of inflammatory status in pJIA. These findings warrant further exploration and validation in a large cohort. Considering the importance of immunopathology classification of synovial biopsy, future studies could investigate the potential of our gene classifiers to predict immunopathology categories of synovial biopsy samples from pJIA patients.

## Data availability statement

The original contributions presented in the study are publicly available. RA data can be found here: https://www.ebi.ac.uk/biostudies/arrayexpress/studies/E-MTAB-13361. pJIA data can be found here: https://www.ebi.ac.uk/biostudies/arrayexpress/studies/E-MTAB-14035.

## Ethics statement

The studies involving humans were approved by Local Ethics Committee from Sao Paulo University-CAPPesq (#51178115.1.0000.0068). The studies were conducted in accordance with the local legislation and institutional requirements. The participants provided their written informed consent to participate in this study.

## Author contributions

BH: Conceptualization, Data curation, Formal analysis, Investigation, Methodology, Visualization, Writing – original draft. LS: Conceptualization, Investigation, Methodology, Writing – original draft. SR: Conceptualization, Investigation, Writing – review & editing. MP: Conceptualization, Writing – review & editing, Investigation. VC: Conceptualization, Investigation, Methodology, Writing – review & editing. DD: Conceptualization, Writing – review & editing. CF: Conceptualization, Writing – review & editing. RP: Conceptualization, Funding acquisition, Methodology, Project administration, Resources, Supervision, Writing – review & editing. EB: Conceptualization, Project administration, Supervision, Writing – review & editing.

## References

[1] Ribeiro SCCR Sales LP Fernandes AL Perez MO Takayama L Caparbo VF. Bone erosions associated with systemic bone loss on HR-pQCT in women with longstanding polyarticular juvenile idiopathic arthritis. Semin Arthritis Rheum. (2023) 63:152247. doi: 10.1016/J.SEMARTHRIT.2023.152247 37595510

[2] Petty RE Southwood TR Manners P Baum J Glass DN Goldenberg J. International League of Associations for Rheumatology classification of juvenile idiopathic arthritis: second revision, Edmonton. J Rheumatol. (2001) 31(2):390–2. Available at: https://www.jrheum.org/content/31/2/390.full.pdf. 14760812

[3] Oberle EJ Harris JG Verbsky JW. Polyarticular juvenile idiopathic arthritis - epidemiology and management approaches. Clin Epidemiol. (2014) 6:379–93. doi: 10.2147/CLEP.S53168 PMC4216020 25368531

[4] Lin YT Wang CT Gershwin ME Chiang BL. The pathogenesis of oligoarticular/polyarticular vs systemic juvenile idiopathic arthritis. Autoimmun Rev. (2011) 10:482–9. doi: 10.1016/J.AUTREV.2011.02.001 21320644

[5] Guilliams M Mildner A Yona S. Immunity review developmental and functional heterogeneity of monocytes. Immunity (2018) 49(4):595–613. doi: 10.1016/j.immuni.2018.10.005 30332628

[6] Klimek E Mikołajczyk T Sulicka J Kwas¨ny-Krochin B Korkosz M Osmenda G. Blood monocyte subsets and selected cardiovascular risk markers in rheumatoid arthritis of short duration in relation to disease activity. BioMed Res Int. (2014) 2014:1–10. doi: 10.1155/2014/736853 PMC4122153 25126574

[7] Schmidt T Dahlberg A Berthold E Król P Arve-Butler S Rydén E. Synovial monocytes contribute to chronic inflammation in childhood-onset arthritis via IL-6/STAT signalling and cell-cell interactions. Front Immunol. (2023) 14:1190018. doi: 10.3389/FIMMU.2023.1190018 37283752 PMC10239926

[8] Throm AA Moncrieffe H Orandi AB Pingel JT Geurs TL Miller HL. Identification of enhanced IFN-γ signaling in polyarticular juvenile idiopathic arthritis with mass cytometry. JCI Insight. (2018) 3:1–14. doi: 10.1172/JCI.INSIGHT.121544 PMC6129135 30089725

[9] Griffin TA Barnes MG Ilowite NT Olson JC Sherry DD Gottlieb BS. Gene expression signatures in polyarticular juvenile idiopathic arthritis demonstrate disease heterogeneity and offer a molecular classification of disease subsets. Arthritis Rheum. (2009) 60:2113–23. doi: 10.1002/ART.24534 PMC2741130 19565504

[10] Sales LP Hounkpe BW Perez MO Caparbo VF Domiciano DS Borba EF. Transcriptomic characterization of classical monocytes highlights the involvement of immuno-inflammation in bone erosion in Rheumatoid Arthritis. Front Immunol. (2023) 14:1251034/BIBTEX. doi: 10.3389/fimmu.2023.1251034 37868981 PMC10588645

[11] Hounkpe BW Chenou F De Lima F De Paula EV. HRT Atlas v1.0 database: redefining human and mouse housekeeping genes and candidate reference transcripts by mining massive RNA-seq datasets. Nucleic Acids Res. (2020) 49(D1):D947–55. doi: 10.1093/nar/gkaa609 PMC7778946 32663312

[12] Dobin A Davis CA Schlesinger F Drenkow J Zaleski C Jha S. STAR: Ultrafast universal RNA-seq aligner. Bioinformatics. (2013) 29:15–21. doi: 10.1093/bioinformatics/bts635 23104886 PMC3530905

[13] Love MI Huber W Anders S. Moderated estimation of fold change and dispersion for RNA-seq data with DESeq2. Genome Biol 2014 15:12. (2014) 15:1–21. doi: 10.1186/S13059-014-0550-8 PMC4302049 25516281

[14] Gu Z Eils R Schlesner M. Complex heatmaps reveal patterns and correlations in multidimensional genomic data. Bioinformatics. (2016) 32(18):2847–9. doi: 10.1093/bioinformatics/btw313 27207943

[15] Reich M Liefeld T Gould J Lerner J Tamayo P Mesirov JP. GenePattern 2.0 [2]. Nat Genet. (2006) 38:500–1. doi: 10.1038/ng0506-500 16642009

[16] Fang Z Liu X Peltz G. GSEApy: a comprehensive package for performing gene set enrichment analysis in Python. Bioinformatics. (2023) 39:1–3. doi: 10.1093/BIOINFORMATICS/BTAC757 PMC9805564 36426870

[17] Shannon P Markiel A Ozier O Baliga NS Wang JT Ramage D. Cytoscape: A software Environment for integrated models of biomolecular interaction networks. Genome Res. (2003) 13:2498–504. doi: 10.1101/gr.1239303 PMC403769 14597658

[18] Ochoa D Hercules A Carmona M Suveges D Baker J Malangone C. The next-generation Open Targets Platform: reimagined, redesigned, rebuilt. Nucleic Acids Res. (2023) 51:D1353–9. doi: 10.1093/NAR/GKAC1046 PMC9825572 36399499

[19] Luck K Kim DK Lambourne L Spirohn K Begg BE Bian W. A reference map of the human binary protein interactome. Nature. (2020) 580:402–8. doi: 10.1038/s41586-020-2188-x PMC7169983 32296183

[20] Culemann S Grüneboom A Krönke G. Origin and function of synovial macrophage subsets during inflammatory joint disease. Adv Immunol. (2019) 143:75–98. doi: 10.1016/BS.AI.2019.08.006 31607368

[21] Elhai M Bazeli R Freire V Feydy A Drapé JL Quartier P. Radiological peripheral involvement in a cohort of patients with polyarticular juvenile idiopathic arthritis at adulthood. J Rheumatol. (2013) 40:520–7. doi: 10.3899/JRHEUM.121013 23418383

[22] Spelling P Bonfa E Caparbo VF Pereira RMR. Osteoprotegerin/RANKL system imbalance in active polyarticular-onset juvenile idiopathic arthritis: a bone damage biomarker? Scand J Rheumatol. (2008) 37:439–44. doi: 10.1080/03009740802116224 18802807

[23] Jang DI Lee AH Shin HY Song HR Park JH Kang TB. The role of tumor necrosis factor alpha (TNF-α) in autoimmune disease and current TNF-α Inhibitors in therapeutics. Int J Mol Sci. (2021) 22:2719. doi: 10.3390/IJMS22052719 33800290 PMC7962638

[24] Jung SM Kim WU. Targeted immunotherapy for autoimmune disease. Immune Netw. (2022) 22:1–23. doi: 10.4110/in.2022.22.e9 PMC8901705 35291650

[25] Fan K Dai J Wang H Wei H Cao Z Hou S. Treatment of collagen-induced arthritis with an anti-osteopontin monoclonal antibody through promotion of apoptosis of both murine and human activated T cells. Arthritis Rheum. (2008) 58:2041–52. doi: 10.1002/ART.23490 18576331

[26] Petrow PK Hummel KM Jo¨ J Schedel J Franz JK Klein CL. EXPRESSION OF OSTEOPONTIN MESSENGER RNA AND PROTEIN IN RHEUMATOID ARTHRITIS effects of osteopontin on the release of collagenase 1 from articular chondrocytes and synovial fibroblasts. Arthritis Rheum. (2000) 43:1597–605. doi: 10.1002/1529-0131 10902765

[27] Gao X Jia G Guttman A Arron JR Khanna D Ramalingam Correspondence TR. Osteopontin links myeloid activation and disease progression in systemic sclerosis. Cell Rep Med. (2020) 1:100140. doi: 10.1016/j.xcrm.2020.100140 33294861 PMC7691442

[28] Giannos P Triantafyllidis KK Giannos G Kechagias KS. SPP1 in infliximab resistant ulcerative colitis and associated colorectal cancer: an analysis of differentially expressed genes. Eur J Gastroenterol Hepatol. (2022) 34:598–606. doi: 10.1097/MEG.0000000000002349 35102110

[29] Xu C Wu Y Liu N. Osteopontin in autoimmune disorders: current knowledge and future perspective. Inflammopharmacology. (2022) 30:385–96. doi: 10.1007/s10787-022-00932-0 35235108

[30] Li X O’Regan AW Berman JS. IFN-γ Induction of osteopontin expression in human monocytoid cells. Journal of Interferon & Cytokine Research. (2004) 23:259–65. doi: 10.1089/107999003321829971 12804068

[31] Saliba J Coutaud B Solovieva V Lu F Blank V. Regulation of CXCL1 chemokine and CSF3 cytokine levels in myometrial cells by the MAFF transcription factor. J Cell Mol Med. (2019) 23:2517–25. doi: 10.1111/JCMM.14136 PMC6433675 30669188

[32] Renoux F Stellato M Haftmann C Kania G Boyman O Correspondence OD. The AP1 transcription factor fosl2 promotes systemic autoimmunity and inflammation by repressing treg development. Cell Reports. (2020) 31:1–15. doi: 10.1016/j.celrep.2020.107826 32610127

[33] Zenz R Eferl R Scheinecker C Redlich K Smolen J Schonthaler HB. Activator protein 1 (Fos/Jun) functions in inflammatory bone and skin disease. Arthritis Res Ther. (2008) 10:1–10. doi: 10.1186/ar2338 PMC2374460 18226189

[34] Zawadzke LE Bugg TD H Walsh CT Strominger JL Ito E Threnn RH. c-fos: a key regulator of osteoclast-macrophage lineage determination and bone remodeling. Sci (1979). (1994) 266:443–8. doi: 10.1126/SCIENCE.7939685 7939685

[35] Luukkonen J Hilli M Nakamura M Ritamo I Valmu L Kauppinen K. Osteoclasts secrete osteopontin into resorption lacunae during bone resorption. Histochem Cell Biol. (2019) 151:475–87. doi: 10.1007/s00418-019-01770-y PMC6542781 30637455

[36] Sugioka K Yoshida K Murakami J Itahashi M Mishima H Nishida T. Inhibition by epigallocatechin gallate of IL-1–induced urokinase-type plasminogen activator expression and collagen degradation by corneal fibroblasts. Invest Ophthalmol Vis Sci. (2019) 60:2895–903. doi: 10.1167/IOVS.19-27306 31266061

[37] Lee KH Choi EY Koh SA Kim MK Jang BI Kim SW. IL-1β-stimulated urokinase plasminogen activator expression through NF-κB in gastric cancer after HGF treatment. Oncol Rep. (2014) 31:2123–30. doi: 10.3892/OR.2014.3086 24626561

[38] Thornton S Raghu H Cruz C Frederick MD Palumbo JS Mullins ES. Urokinase plasminogen activator and receptor promote collagen-induced arthritis through expression in hematopoietic cells. Blood Adv. (2017) 1:545. doi: 10.1182/BLOODADVANCES.2016004002 29296974 PMC5728599

[39] Busso N Péclat V So A Sappino AP. Plasminogen activation in synovial tissues: differences between normal, osteoarthritis, and rheumatoid arthritis joints. Ann Rheum Dis. (1997) 56:550–7. doi: 10.1136/ARD.56.9.550 PMC1752434 9370880

[40] Enocsson H Lukic T Ziegelasch M Kastbom A. Serum levels of the soluble urokinase plasminogen activator receptor (suPAR) correlates with disease activity in early rheumatoid arthritis and reflects joint damage over time. Trans Res. (2021) 232:142–9. doi: 10.1016/J.TRSL.2021.02.007 33582243

[41] Rosenberg AM. Do we need a new classification of juvenile idiopathic arthritis? Clin Immunol. (2020) 211:108298. doi: 10.1016/J.CLIM.2019.108298 31706029

[42] Eng SWM Duong TT Rosenberg AM Morris Q Yeung RSM. The biologic basis of clinical heterogeneity in juvenile idiopathic arthritis. Arthritis Rheumatol. (2014) 66:3463–75. doi: 10.1002/art.38875 PMC4282094 25200124

[43] Rezaei E Hogan D Trost B Kusalik AJ Boire G Cabral DA. Associations of clinical and inflammatory biomarker clusters with juvenile idiopathic arthritis categories. Rheumatology. (2020) 59:1066–75. doi: 10.1093/RHEUMATOLOGY/KEZ382 32321162

