# Supplementary material for: Transcriptomic signatures of classical monocytes reveal pro-inflammatory modules and heterogeneity in polyarticular juvenile idiopathic arthritis
Source: Front Immunol. 2024 May 21;15:1400036. doi: 10.3389/fimmu.2024.1400036 (PMC11148224; doi:10.3389/fimmu.2024.1400036)
Supplement: Supplementary file 1 [file DataSheet_1.docx]

**Supplementary figures**

Transcriptomic Signatures of Classical Monocytes Reveal Pro-Inflammatory Modules and Heterogeneity in Polyarticular Juvenile Idiopathic Arthritis

Bidossessi W. Hounkpe¹^†^, Lucas P. Sales¹^†^, Surian C.R Ribeiro¹, Mariana O. Perez¹, Valéria F. Caparbo¹, Diogo Souza Domiciano¹, Camille P. Figueiredo¹, Rosa M.R. Pereira¹ ⸆, Eduardo Ferreira Borba¹*

^1^Hospital das Clínicas HCFMUSP, Faculdade de Medicina, Universidade de São Paulo, São Paulo, Brasil

^†^These authors contributed equally to this work

*** Correspondence:**Eduardo Ferreira Borba
eduardo.borba@hc.fm.usp.br


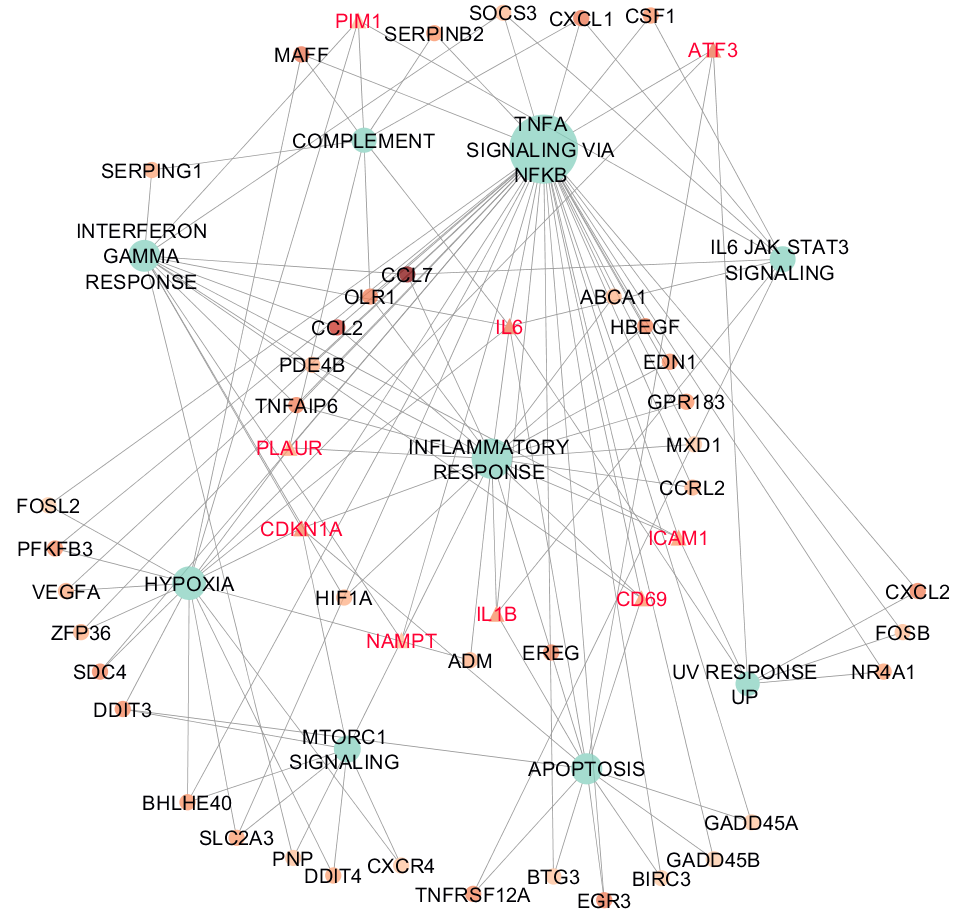


Figure S1: Connected network of the significant gene sets identified in pJIA. Hub genes are labeled in red and pathways are indicated in green. The size of the dots of pathway correspond to their betweenness connectivity. The larger the size, the greater the importance of the pathway in the network. The intensity of the red color indicates the magnitude of the fold change of expression.


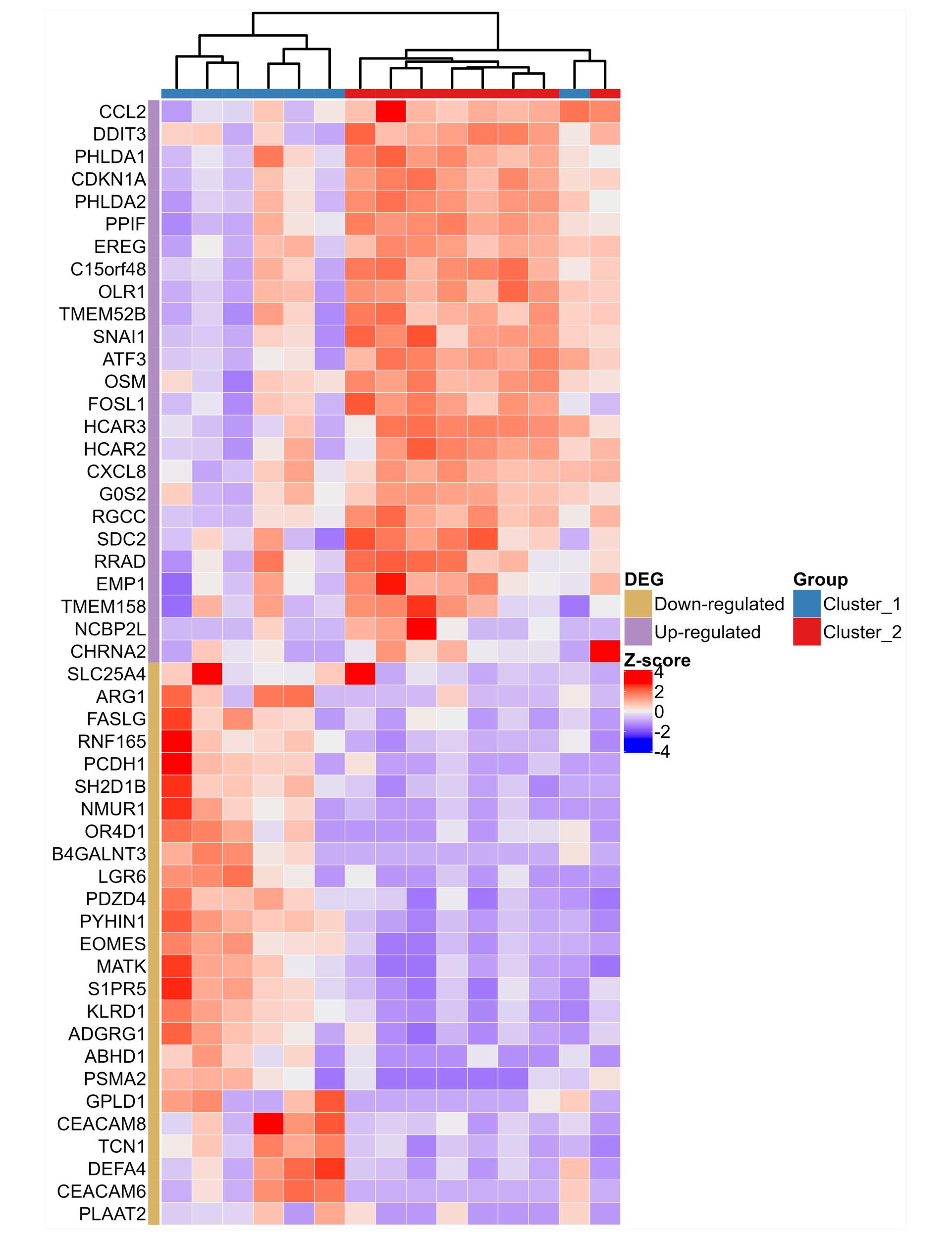


Figure S2: Heatmap showing expression patterns of the top-50 ranked DEGs of the comparison of cluster 2 *vs* cluster 1. pJIA patients are stratified in two homogenous clusters


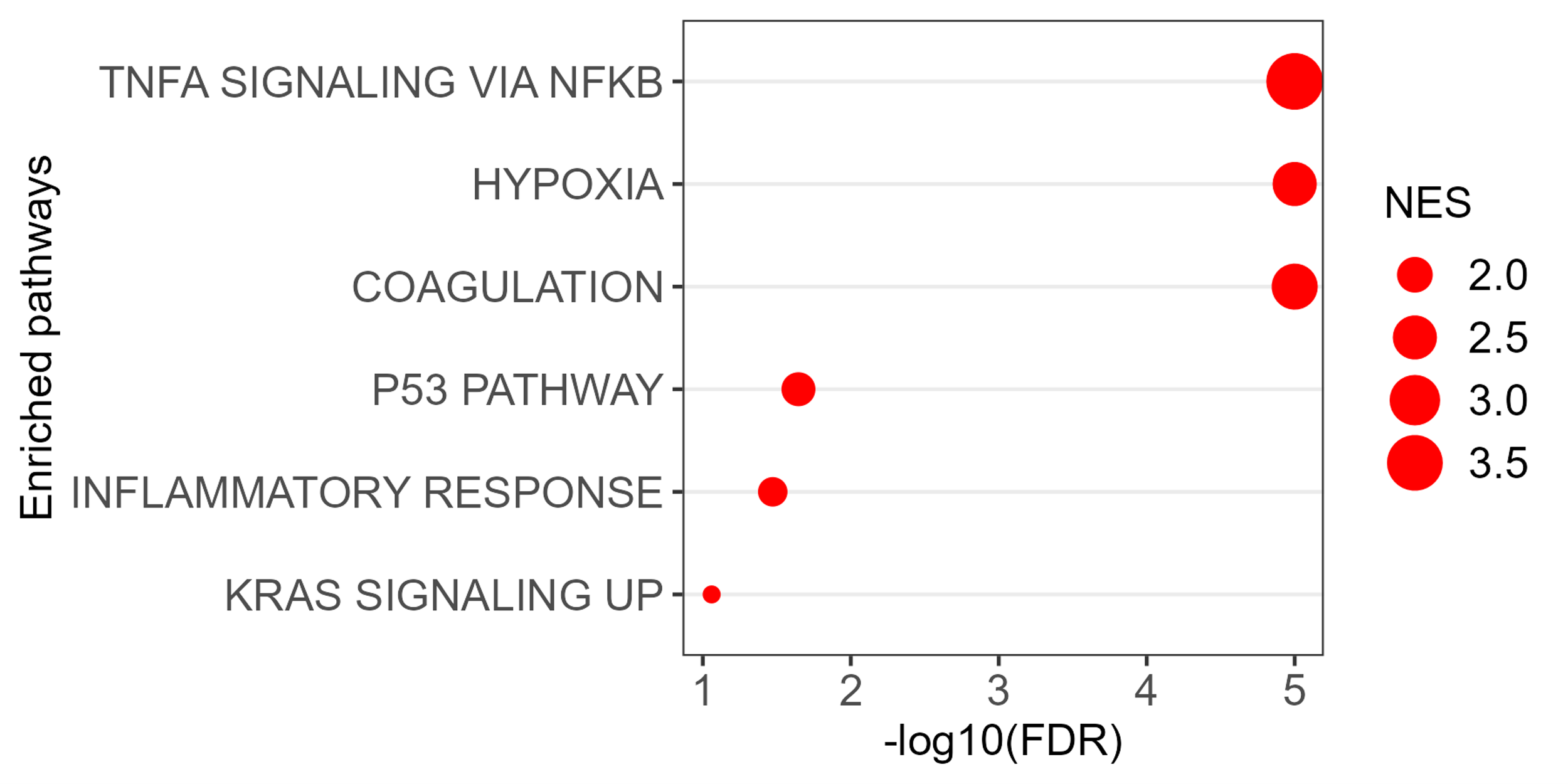


Figure S3: Dot plot of functional analysis performed using the hallmark gene sets (GSEA) showing the enrichment of pathways associated with the activation of immuno-inflammation in cluster 2. Dot size represents the normalized GSEA enrichment score (NES) and the X axis indicated the FDR.

GSEA: Gene Set Enrichment Analysis; FDR: False Discovery Rate; NES: Normalized Enrichment Score.
